# Protozoa populations are ecosystem engineers that shape prokaryotic community structure and function of the rumen microbial ecosystem

**DOI:** 10.1038/s41396-021-01170-y

**Published:** 2021-12-09

**Authors:** Ronnie Solomon, Tanita Wein, Bar Levy, Shahar Eshed, Rotem Dror, Veronica Reiss, Tamar Zehavi, Ori Furman, Itzhak Mizrahi, Elie Jami

**Affiliations:** 1 Department of Ruminant Science, Institute of Animal Sciences, Agricultural Research Organization, Volcani Center, Rishon LeZion, Israel; 2 Institute of Natural Sciences, Department of Life Sciences, Ben-Gurion University of the Negev, Beersheba, Israel; 3 Department of Molecular Genetics, Weizmann Institute of Science, Rehovot, Israel; 4 The Mina and Everard Goodman Faculty of Life Sciences, Bar-Ilan University, Ramat Gan, Israel

**Keywords:** Microbial ecology, Food webs

## Abstract

Unicellular eukaryotes are an integral part of many microbial ecosystems where they interact with their surrounding prokaryotic community—either as predators or as mutualists. Within the rumen, one of the most complex host-associated microbial habitats, ciliate protozoa represent the main micro-eukaryotes, accounting for up to 50% of the microbial biomass. Nonetheless, the extent of the ecological effect of protozoa on the microbial community and on the rumen metabolic output remains largely understudied. To assess the role of protozoa on the rumen ecosystem, we established an in-vitro system in which distinct protozoa sub-communities were introduced to the native rumen prokaryotic community. We show that the different protozoa communities exert a strong and differential impact on the composition of the prokaryotic community, as well as its function including methane production. Furthermore, the presence of protozoa increases prokaryotic diversity with a differential effect on specific bacterial populations such as Gammaproteobacteria, *Prevotella* and *Treponema*. Our results suggest that protozoa contribute to the maintenance of prokaryotic diversity in the rumen possibly by mitigating the effect of competitive exclusion between bacterial taxa. Our findings put forward the rumen protozoa populations as potentially important ecosystem engineers for future microbiome modulation strategies.

## Introduction

Microbial community assemblages are determined by both abiotic and biotic factors driven by the environmental conditions and a complex network of microbial interactions between diverse microorganisms.

Microbial eukaryotes—such as protists—are ubiquitous in a wide range of environments and play a pivotal role in regulating microbial community structure and function as well as their physicochemical environment (e.g., [1]). Protist-bacteria interactions range from mutualistic (e.g., metabolic exchange or scavenging of toxic compounds) [2–4], to antagonistic interplay that mainly comprises predation [1–3]. Protists were shown to positively interact with bacteria and archaea. This is exemplified by various interspecies exchanges of metabolites with the surrounding prokaryotic community contributing to their respective fitness [4, 5], as well as evidence of physical interaction with prokaryotic cells localized inside, or surface attached to the protozoa [2, 6]. As predators, protists are considered a major cause of bacterial mortality in microbial ecosystems, where they exert a top-down control that was shown to greatly impact surrounding prey species. Protist-predation further leads to changes at the microbial community structure level, as well as at the single-cell level, which can promote changes in bacterial morphology and evolution [1, 7–10].

In host-associated microbial communities, protists were suggested to play a beneficial role for their host. For example, in the rhizosphere of plants, predation by protists promotes changes in microbial composition that accelerates nutrient cycling and supports the removal of pathogenic species [11–13]. Though less evidence exists regarding mammalian hosts, the protist community was suggested to confer protection to the host via immune mediated response against pathogens [14, 15]. Additionally, based on population analysis of the microbiome in the human gut, the presence of *Blastocystis* was suggested to play a critical role in maintaining bacterial diversity in the gut microbiome [15]. Such evidence suggests that protists not only play a crucial role for microbial community assemblages, but may also have a direct effect on the host species and alterations of these interactions have the potential to greatly affect microbial ecosystems. Nonetheless, the complex role of protists in diverse environments including animal-associated microbiomes remains largely understudied.

One of the most densely populated gut environments is the upper digestive tract of ruminants, termed rumen. The rumen hosts one of the most complex microbial communities comprising bacteria, archaea and microbial eukaryotes, the latter being dominated by ciliate protozoa [16]. Ciliate protozoa species are ubiquitously found in the rumen, and are estimated to encompass between 25% and 50% of the microbial biomass [17–19]. The protozoa population, like bacteria and archaea, encompass a large array of diverse species in the rumen environment [20], historically characterized by different morphologies and sizes, with species larger than 100 μm to smaller 10 μm in length [21]. Rumen protozoa are part of a complex microbial community responsible for the breakdown of plant feed into digestible molecules for the animal and accordingly, ruminant productivity has been, in recent years, tightly linked with the rumen microbial community composition [22–26]. Unlike bacteria, protozoa are not obligatory in the rumen and can be removed with no apparent ill-effect to the host, via defaunation [18]. Defaunations thus allows for controlled in-vivo experiments in which the role of the protozoa can be evaluated by either addition or subtraction of the whole ciliate protozoa community or specific subpopulations [18]. Such experiments revealed that the removal of protozoa from the rumen carries a tremendous effect on the production of metabolic end-products and nitrogen metabolism and that different protozoa taxa contribute differently to the metabolic aspects of the rumen ecosystem and animal physiology [18, 27–31]. Specifically, the absence of protozoa was shown to decrease methane emission in defaunated animals. This observation suggests a metabolic interaction between protozoa and methanogenic archaea, the sole producers of methane in the rumen. Protozoa are known for their production of hydrogen while the majority of methanogens in the rumen produce methane via the hydrogenotrophic pathway. This notion is reinforced by observations of physical association between protozoa and methanogens [4, 32–34]. In addition, the presence of protozoa in the rumen was shown to decrease the overall protein supply to the animal in-vivo [18]. This may be attributed to the predatory behavior of protozoa, as ruminant protein supply largely depends on bacterial degradation in the abomasum [18, 35]. Estimates suggest that between 0.1% of rumen bacteria are digested by the rumen protozoal population every minute at a protozoa concentration of 2 × 10^6^ protozoa cells per ml [36]. Defaunation experiments thus suggest a central role of protozoa in the rumen ecosystem, including their effect on the prokaryotic community and the host animal.

Despite the perceived importance of protozoa communities on the rumen ecosystem and our environment, the direct effect of protozoa community and its composition on the prokaryotic community structure and function was never examined. In this study, we aimed to assess the role of rumen protozoa in shaping prokaryotic community structure and function. We hypothesize that various protozoa populations exert differential effects on the native prokaryotic community in the rumen. To this end, freshly sampled rumen prokaryotic community was exposed to rumen protozoa sub-communities in order to characterize the resulting metabolic output as well as the microbial prokaryotic community dynamics. Our findings suggest that rumen protozoa play a central role in defining characteristics of the rumen ecosystem shaping rumen microbiome structure and metabolism.

## Materials and methods

### Animal handling and sampling

The experimental procedures used in this study were approved by the Faculty Animal Policy and Welfare Committee of the Agricultural Research Organization Volcani Research Center approval no. 737/17 IL, in accordance with the guidelines of the Israel Council for Animal Care.

Rumen fluid was sampled 2 h after feeding, from three cows kept under the same high fiber diet (70% roughage and 30% grains), which is the standard diet for cows during the dry period in our institute [37] and according to NRC 2001 requirements [38], for at least two months. After sampling, the rumen fluid was immediately transferred to an oxygen-free environment in an anaerobic glove box for further processing.

### Protozoa separation

In order to obtain different populations of protozoa, the rumen samples underwent a series of size filtration and washings similar to the procedure performed in [32]. Briefly, the rumen fluid was mixed in a 1:1 ratio with anaerobic Coleman buffer warmed at 39 °C [20], and incubated in a separating funnel for 1 h under anaerobic conditions at 39 °C. The settled protozoa fraction was transferred to a fresh tube with warm Coleman buffer. Prior to filtration a subset of the whole protozoa community was put aside and represents the all protozoa group in the study. The rest of the protozoa underwent consecutive filtration using nylon net filters (Merck Millipore, Darmstadt, Germany) of different sizes (i.e., 100, 60, 40, 10 μm). The retentate on each filter and the filtrate of the last 10 μm filtering were then washed with an anaerobic Coleman buffer warmed at 39 °C [20]. A subset of each fraction was taken for counting under light microscopy in order to be able to inoculate the microcosms with the same number of protozoa. Paraformaldehyde at a final volume of 4% was added to 3 drops of 10 μl of each of the fractions and the average number obtained represented the overall protozoa concentrations for each of the fractions. A second subset of the washed fractions was kept frozen for further 18S rRNA amplicon sequencing and biomass quantification via the Bradford protein assay [39]. The prokaryotic community was obtained from the upper phase obtained during the protozoa sedimentation process and was centrifuged once at 500 × *g* to remove potential remaining protozoa. Only the upper half of the supernatant was used to minimize contamination of protozoa after centrifugation. It is important to note that the methodological setup of this study focuses on the liquid-associated fraction of the prokaryotic community as these were suggested to more likely be predated than the particulate-associated prokaryotes [28].

### Biomass quantification

Protozoa of each fraction were twice washed using anaerobic phosphate-buffered saline and centrifuged at 4700 × *g* for 15 min. Cells were lysed using beat-beating performed three times for 30 s at maximun speed at 4 °C. The lysate was centrifuged at 4700 × *g* for 15 min and the supernatant was used to quantify protein content using the Bradford protein assay [39]. A Bradford calibration curve was generated by various BSA concentrations, ranging between 0 and 1 mg/ml. The non-linear equation obtained by the calibration was used to quantify protein concentrations of all protozoa samples.

### Microcosm preparation

The prokaryotic community was distributed evenly in 20 ml anaerobic screw-cap glass tubes equilibrated in the anaerobic glove box. The rumen fluid containing the prokaryotic community was inoculated with 100 mg of ground feed of the same composition the cows received as substrate. The protozoa fractions were centrifuged twice and concentrated in order to inoculate the microcosms with the smallest amount of volume to minimize the carryover of additional ruminal factors that might affect our experiment (150–250 μl, up to 2.5% of the final volume). The overall volume of each microcosm was 10 ml containing 10^4^/ml of protozoa from each community, one treatment with the full native protozoa community (similarly adjusted to 10^4^/ml cells) and one treatment without protozoa was named ‘protozoa-free’. The number of protozoa was chosen to reflect the typical abundance of protozoa in the rumen and was also based on a previous experiment showing that this number shows a visible change in methane production. Furthermore, we opted for a similar number of protozoa in order to allow for a direct comparison between the fractions. The requirement for such protozoa numbers hindered our ability to always obtain the aimed triplicates for all the cows and fractions, thus some groups were performed with two replicates (cow 1; *P* < 10, cow 2; all protozoa, P-100). One additional all protozoa community was removed from the analyses following as it showed contamination with a species of Betaproteobacteriales, not native to the rumen. The microcosms were incubated for 96 h tilted at 20°. Methane quantification was performed after each day for four days. After methane quantification, 5 ml of the upper fraction of the microcosms was removed and kept frozen at −80 °C for quantification of volatile fatty acids (VFAs) and sequencing of the prokaryotic community. The microcosm was complemented with 5 ml of medium M [20], and incubated further. All the procedures were performed under anaerobic conditions.

### Metabolites quantification

Methane and VFA quantification was performed following the protocol from Shabat et al. [22]. For methane, the incubated samples were removed from incubation and directly placed into the Gas Chromatography (GC) autosampler ten samples at a time. Samples of 0.250 ml of gas from the headspace of the tubes were injected into a 182.88 cm × 0.3175 cm × 2.1 mm packed Supelco analytical-45/60 Molecular sieve 5 A column (Supelco Inc., Bellefonte, PA, USA) with helium carrier gas set to a flow rate of 10 ml min^–1^ and an oven temperature of 200 °C. The oven temperature remained steady for a total run time of 5 min. A standard curve was generated using pure methane gas. After the daily measurement 5 ml of fluid from each microcosm was removed for VFA quantification and microbiome analysis. For VFA measurement, the removed fluid was centrifuged at 10,000 × *g* in order to first separate the microbial community from the incubated fluid. The supernatant was transferred to a new tube and the pellet was used for further DNA extraction. Eight hundred microliters of the supernatant was mixed with 200 μl of 25% metaphosphoric acid solution (w/v in DDW) followed by 1 min vortex and then incubated at 4 °C for 30 min. The samples were then centrifuged for 15 min at 10,000 g and the supernatant was removed into new tubes, then 250 μl methyl tert-butyl ether (Sigma-Aldrich) was added and the tubes were vortexed for 30 s. Another cycle of centrifugation was performed for 1 min at 10,000 g. The upper phase, which contained methyl tert-butyl ether +short chain fatty acids, was analyzed using an Agilent 7890B GC system (Agilent Technologies, Santa Clara, CA, USA) with a Flame ionization detector. The temperatures at the inlet and detector were 250 °C and 300 °C, respectively. Aliquots (1 μl) were injected with a split ratio of 1: 11 into a 30 m × 0.32 mm × 0.25 μm ZEBRON ZB-FFAP column (Phenomenex, Torrance, CA, USA) with helium carrier gas set to a flow rate of 2.4 ml min^–1^ and initial oven temperature of 100 °C. The oven temperature was held constant at the initial temperature for 5 min, and thereafter increased at 10 °C min^–1^ to a final temperature 125 °C, and a final run time of 12.5 min. Individual injections of each pure VFA was performed in order to identify their retention in the column and a calibration curve was generated by preparing an equimolar solution of all the VFA and serially diluting it from 100 to 0.1 mM.

### DNA extraction

DNA extraction was performed as previously described [40]. In brief, cells were lysed by bead disruption using Biospec Mini-Beadbeater-16 (Biospec, Bartlesville, OK, USA) at 3000 RPM for 3 min with phenol followed by phenol/chloroform DNA extraction. The final supernatant was precipitated with 0.6 volume of isopropanol and resuspended overnight in 50–100 μl TE buffer (10 mM Tris-HCl, 1 mM EDTA), then stored at 4 °C for short-term use, or archived at −80 °C.

### Illumina amplicon sequencing

The V4 region of 16S rRNA was amplified by PCR from DNA extracts using barcoded primers 515F 5′-CCTACGGGAGGCAGCAG-3′ and 806rcbR 5′-CCGTCAATTCMTTTRAGT-3′ [41]. The barcoded samples were pooled, sequenced in a MiSeq flow cell (Illumina) for 250 cycles from one end of the fragment and analyzed with Casava 1.8. Amplicon sequencing was performed for the 18S rRNA of the fractionated ruminal samples using primers specifically designed for ciliates taken from Tapio et al. (2016) [42] with the following sequences: CiliF (5′-CGATGGTAGTGTATTGGAC-3′) and CiliR (5′-GGAGCTGGAATTACCGC-3′). Ruminal DNA samples were treated as follows: 20 ng of DNA was used in a 25 μl PCR amplification with primers, using PrimeStar Max DNA Polymerase (Takara) for 20 cycles. The PCR reaction was purified using AmpureXP beads, and then a second PCR was performed using the Fluidigm Access Array primers for Illumina to add the adapter and index sequences. For this reaction 2 μl of the first PCR were amplified in a 10 μl reaction for 10 cycles. The PCR product was purified using AmpureXP beads and the concentrations were measured by Qubit. The samples were pooled, run on a DNA D1000 screentape (Agilent) to check for correct size and for the absence of primer-dimers product. The pool was then sequenced on the MiSeq (Illumina), using the MiSeq V2-500 cycles sequencing kit.

### Data analysis

The relative contribution of protozoa to metabolites production was done by assessing the difference between each microcosms from a specific cow to the corresponding protozoa-free microcosms and subsequently normalized based on protein quantification of the washed protozoa fractions. Differential production of metabolites was assessed using ANOVA two-way performed on transformed data (using aligned rank transformation ART [43]), in order to account for interactions between the different protozoa fractions and the effect of the individual cows. When the analysis indicated a significant difference between the groups, a *post-hoc* Aligned Rank Transform Contrasts (ART-C) was performed to determine which paired groups differed from each other using the Artool package in R [43, 44].

Downstream processing of the 16S rRNA data, up to the generation of the amplicon sequence variant table (ASV) was performed in QIIME v.2 [45]. DADA2 was applied to model and correct Illumina-sequencing amplicon errors and clustering of ASVs [46]. Taxonomic assignment for the bacterial 16S rRNA was performed using the pre-trained classifier Silva database [47] (silva_132_16S.97) ASVs from 515F/806R region from QIIME v.2 pipeline. After the generation of the ASV table, singletons/doubletons were removed and subsampling to an even depth of 4000 reads per sample was performed for all subsequent analyses. Alpha and Beta diversity analyses were performed and plotted using the PAleontological STatistics software [48], including principal coordinate analysis (PCOA) using the Bray–Curtis dissimilarity metric and ASV richness, evenness, and Shannon index. Analysis of similarity (ANOSIM) was used to test the significance of the group clustering. Distance-based redundancy analysis (DB-RDA) was performed with the capscale function in the vegan package in R [49] and using the Bray-Curtis distance metric.

Centered log-ratio transformation was performed for the statistical analysis of compositional data, using the ‘compositions’ R package [50]. The analyses were firstly performed on each cow source individually due to the intrinsic differences in community composition between cows using Kruskal–Wallis along with the Wilcoxon test for pairwise comparisons between the groups. When similar observations were observed between the different cows we analyzed the whole dataset was combined and analyzed using ANOVA two-way performed on transformed data (using aligned rank transformation ART [43]), in order to account interaction between the different fraction groups and cow effect [51]. When the analysis indicated a significant difference between the groups, a *post-hoc* Aligned Rank Transform Contrasts (ART-C) was performed to determine which paired groups differed from each other [44] using the Artool package in R. When F values of ANOVAs on aligned responses not of interest did not meet the requirement of being ~0, as recommended by its developer [43], Kruskal-Wallis was performed along with the Wilcoxon test for pairwise comparisons between the groups.

For all the analyses, *p* values of <0.05 after false discovery rate correction via the Benjamini Hochberg procedure were considered significant, unless otherwise stated in the text or figure. Statistical tests and data analysis across the different fractions were performed in R version 3.5.3 [52]. Multiple sequence alignment was performed using Multiple Alignment Fast Fourrier transform, using the default parameters. The resulting multiple sequence alignment was used for the reconstruction of a maximum-likelihood phylogenetic tree using IQTree [53], with LG model and 1000 bootstrap replicates. The phylogenetic tree was visualized using iTOL [54].

## Results

### Experimental design

**Fig. 1:**
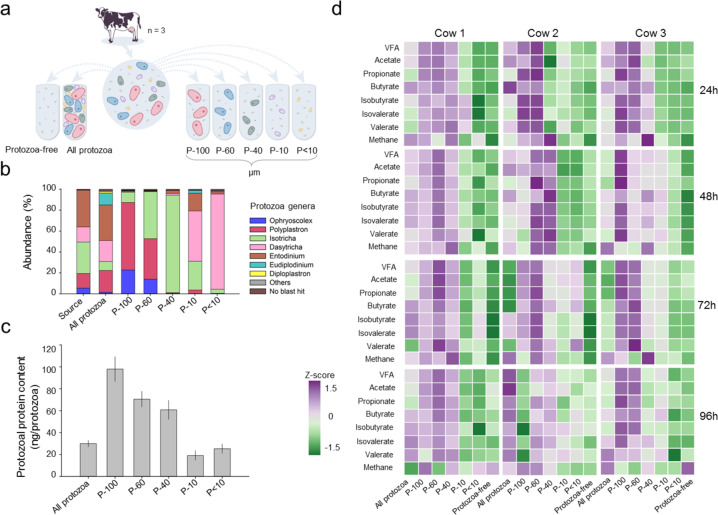
Experimental design and metabolic output of microcosms incubated with different protozoa communities. **a** Experimental setup of the microcosm experiments. The rumen microbial community of three cows were sampled and separated from protozoa cells. This was conducted either for all protozoa or according to the protozoa size indicated by P-100, P-60, P-40, P-10, P < 10 representing the different filters used for the separation. Subsequently, the prokaryotic community was incubated with the different protozoa populations. **b** The genus-level distribution of protozoa in the source community sampled from the animals and the different fractions obtained. **c** Average protozoa biomass in each protozoa fraction obtained by measuring the protein content of each protozoa fraction. **d** Metabolic output of microbial communities incubated with different protozoa communities and without protozoa. Metabolites were measured every 24 h in microbial communities from three cows (*n* = 3 for each cow) for 96 h that were incubated with different protozoa-size populations. Each row is represented by the *z*-score for each individual metabolite (for original data see Supplementary Fig. S1).

To study the effect of protozoa on the metabolic output and the prokaryotic community of the cow rumen, we performed a series of in-vitro microcosm experiments. The experiments were initiated by sampling the rumen fluid of three cows that were kept under the same diet for 2 months prior to the experiment. To produce protozoa communities characterized by different taxonomic composition we utilized the fact that different protozoa species have distinguishable sizes and shapes, and used an established approach in which the protozoa are fractionated by size [32, 55]. The protozoa community was fractionated into five fractions representing different protozoa sizes from >100 to <10 µm (Fig. 1a). Using this procedure, we obtained protozoa communities that differ in taxonomic composition as characterized by 18S rRNA amplicon sequencing analysis (Fig. 1b). The sampled ‘source’ protozoa community exhibited a type A composition with *Polyplastron* being a key member of this population type [20]. The taxonomic composition of the protozoa fractions containing all protozoa was highly similar to the composition of the source community directly analyzed from the host animals (Fig. 1b). The fractions P-100 and P-60, were characterized by large protozoa mainly including *Ophryoscolex* and *Polyplastron* genera, with P-60 including *Isotricha*. The P-40 fraction was almost exclusively composed of *Isotricha*. Fractions P-10 and P- < 10 were dominated by *Dasytricha*, and to a lesser extent by *Entodinium*. The *Isotricha* genus was detected in all size fractions albeit in different relative abundances (e.g., between ~93% in P-40 and ~5% in P- < 10) (Fig. 1b).

After having established different protozoa communities, we set forward to measure their effect on an identical prokaryotic community. To this end, we adjusted the total number of protozoa cells for all subpopulations to 10^4^ cells/ml per tube (10^5^ cells overall in 10 ml medium) and exposed them to identical prokaryotic communities. Each protozoa community coming from each of the three cows were reintroduced to their respective native prokaryotic community of the cow they originated from. The resulting microcosms consisted of replicates of each communities along with replicate for the rumen prokaryotic community incubated without protozoa (hereafter referred to as”protozoa-free” microcosms) and a fraction in which the whole protozoa population was reintroduced (hereafter referred to as”all protozoa” microcosms; Fig. 1a). Overall, our experiment included three biological replicates per protozoa sub-community or control (21 experiments) and was conducted on three types of rumen bacterial communities originated from three different cows comprising an overall of 61 microcosms. The microcosms were incubated for 96 hours, and prokaryotic community composition as well as the methane and VFA production was assessed every 24 h.

### Methanogenesis is enriched in specific protozoal sub-communities

We first measured the effect of the different protozoal communities on ecosystem function, which was manifested by fermentation products and methanogenesis. Methane production requires the mutualistic interspecies hydrogen transfer between a hydrogen producer and methanogenic archaea [4, 56]. Our results show a clear and significant enrichment of methanogenesis in the protozoa fraction P-40 (Fig. 1d; Wilcoxon test *p* < 0.01). The P-40 community dominated by *Isotricha* exhibited a 1.5-fold higher methane output compared to fractions P-100, which exhibited the second highest methane production. Furthermore, P-40 protozoa exhibited a ~3-fold higher methane production compared to the protozoa-free microcosms after 48 h (Fig. 1d; Fig. S1). The higher production of methane in the P-40 fraction could still be observed when normalizing the methane of each fraction to the protozoa biomass and methane production of the protozoa-free community introduced in the microcosms. When normalizing per unit of protozoal biomass, P-40 and P-10 exhibited a higher contribution to methane production compared to P-100 and P-60 (ART-C *p* < 0.01; Fig. S2). Overall, the methane production in the small protozoa fractions P-10 and F < -10 was significantly lower than in the large protozoa fractions P-40, P-60, and P-100 (ART-C test *p* < 0.05), but was still significantly higher when compared to the protozoa-free microcosms (ART-C test; *p* < 0.05; Fig. S1, Table S1).

These results corroborate the role of protozoa in increased rumen methanogenesis and highlight the *Isotricha* dominated community as a high methane-producing community.

Production of VFAs was significantly higher in several protozoa communities. Mainly, microcosms incubated with large sized P-100, P-60, and P-40 were significantly higher compared to the protozoa-free community, while the small P-10 and P- < 10 protozoa communities did not exhibit a significant difference with the protozoa-free community (Fig. 1d; Fig. S1, Table S1).

When normalizing the contribution in VFA production to the overall biomass of each protozoa fraction introduced to the microcosms, P-100 was significantly lower than P-60 and the all-protozoa fractions (ART-C, *p* < 0.05; Fig. S2), and not significantly different than P-40 (Fig. S2).

Our results show that the protozoa populations have a distinct effect on the overall metabolic output of the microbial community. While higher VFA production could only be observed microcosms containing a larger protozoa biomass, methane production showed a pattern which suggests that additional factors may be at play.

### Protozoa sub-communities differentially shape prokaryotic community structure

**Fig. 2:**
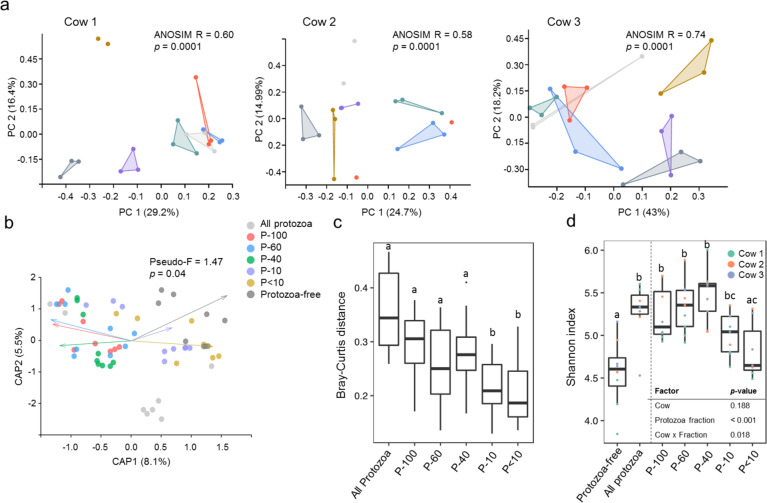
Ecological structure of the prokaryotic community across microcosms. **a** Principal coordinate analysis (PCOA) plot of the microcosms separately plotted according to the cow the microbiome originates from (cow 1–3) and based on Bray-Curtis distance metric at the end of the experiment (96 h). Analysis for all time points of the experiment are in the Supplementary material (Fig. S3). Analysis of similarity (ANOSIM) was performed to assess the protozoa community-mediated discrimination (shown in upper right corner of each plot). **b** Distance-based redundancy analysis (dbRDA) combining all the microcosms from the different cows and fraction at the end of the experiment with a permutational multivariate analysis of variance (PERMANOVA) showing the discrimination between the fraction. Pairwise PERMANOVA between the fractions can be found in the Supplementary material (Table S2). **c** Bray–Curtis distance between the protozoa-free microcosms and microcosms containing different protozoa communities. Pairwise Wilcoxon rank-sum test was used to test for significance and boxes that are not sharing a letter denote significance at *p* < 0.05. **d** Shannon diversity across the different treatments after 96 h of incubation. The table on the lower right side of the plot shows the results of ANOVA two-way performed on transformed data (using aligned ranked transformation [39]) to test cow effect and protozoa fraction effect. Boxes that are not sharing a letter denote a significant difference between the groups at *p* < 0.05.

To study the effect of protozoa on the microbial community structure in the rumen, we analyzed the bacteria and archaea composition with relation to the different protozoa populations in the microcosm, across the 96 h of incubation via amplicon sequencing of the 16S rRNA in each microcosm. Our analysis revealed a clear and a strong causal effect of the distinct protozoa communities on the prokaryotic community structure in all of our biological replicates (microcosms and replicates coming from the different cows; Fig. 2, Fig. S3). Using the pairwise Bray-Curtis distance between the samples, a protozoa community-based discrimination in prokaryotic community structure was already detectable after 24 h and remained stable until the end of the experiment, as observed by the PCOA clustering of the prokaryotic community as a function of the different protozoa populations (Fig. 2a; Fig. S3). Furthermore, replicates inoculated with the same protozoa community were significantly more similar to each other than between the different protozoa communities (Wilcoxon test, *p* < 0.001; Fig. S4a). The individual effect of the protozoa communities on prokaryotic structure was evident in all three rumen prokaryotic communities originating from the different cows, despite the large differences stemming from the individual cows (Fig. S4b; ANOSIM R = 0.98, *p* < 0.001), and dynamics across the days of sampling (Fig. S4c). Notably, the time-dependent difference between 72 h and 96 h was marginal, while the differences in community structure stemming from the different protozoa communities remained stable (Fig. S4c). When combining the samples stemming from all the cows together, DB-RDA confirmed this discrimination (RDA Pseudo-F = 1.47, *p* = 0.042; Fig. 2b), with P-100, P-60, P-40, and all-protozoa fraction being significantly different than the protozoa-free microcosms (Table S2). To further evaluate the strength of change in prokaryotic community structure induced by the different protozoa communities, we compared the Bray-Curtis distance between protozoa-free to protozoa-containing communities, which revealed that the distance was largely dependent on the size of the protozoa (Fig. 2c). Large protozoa cells (P-100, P-60, and P-40) exhibited a higher distance than smaller protozoa sizes (P-10 and P- < 10) compared to the protozoa-free microcosms (Fig. 2c). The largest distance from the protozoa-free population was observed in the fraction containing the native protozoa population despite encompassing a lower average protozoa biomass (i.e., all-protozoa fraction, Fig. 2c).

We further analyzed the effect of the inoculated protozoa communities on the alpha diversity parameters of the prokaryotic communities. While we did not observe a consistent difference in diversity at the first two days of incubation (Fig. S5), over time (after 72 h and 96 h) microcosms incubated with P-100, P-60, and P-40 invariably resulted in a significantly higher Shannon index, species richness and evenness than the protozoa-free community (ART test, *p* < 0.001; Fig. 2d; Fig. S5). Notably, fraction P-40 representing an intermediate protozoa biomass, exhibited the highest prokaryotic diversity in most microcosms compared to the protozoa-free microcosm (Shannon index average; Protozoa-free = 4.58, P-40 = 5.47; *p* < 0.001), driven by a higher ASV richness (Fig. 2d, Fig. S5).

Our results show that the presence of protozoa populations had a strong impact on the rumen microbial ecosystem diversity and that different protozoa communities differentially affect prokaryotic community structure. Protozoa of larger size and overall biomass induced stark alterations in the microbial community structure, while prokaryotic community richness peaked in the intermediate-sized protozoa fractions.

### Protozoa positively affect specific rumen prokaryotic lineages with a strong effect on the enrichment of Gammaproteobacteria

**Fig. 3:**
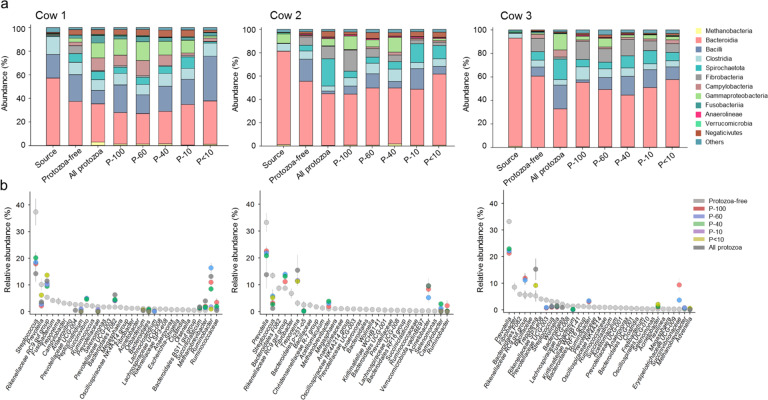
Taxonomic composition across the microcosms. **a** Stack bars displaying the class level relative abundance of taxa across the different protozoa communities, the protozoa-free community and for each cow, the original prokaryotic composition of the samples taken (termed ‘source’). **b** The relative abundance of the different genera in the microbial communities is displayed for each cow. The genera are ordered based on their rank abundance in the protozoa-free microcosms. Color-coding is based on the different protozoa communities added to the prokaryotic community and only genera that were significantly different from the protozoa-free fraction are displayed (*p* < 0.05, on centered log ratio transformed data).

To quantify the effect of protozoa on the abundance of specific prokaryotic species, we analyzed the taxonomic distribution across all samples. The analysis yielded 13 classes, 27 families, and 33 genera that were present on average above 0.5% of the total prokaryotic community in at least one group of replicates and represented between 85% and 97% of the total prokaryotic community. We observed a large expansion of Proteobacteria in all protozoa-containing microcosms, chiefly attributed to Gammaproteobacteria, already observable after 24 h (Fig. 3a; Fig. S6). The increase in Gammaproteobacteria abundance was most pronounced in communities incubated with large protozoa fractions (P-100, P-60, P-40), (ART-C test, *p* < 0.001; Fig. 3a; Fig. S6). Notably, one class of methanogenic archaea, Methanobacteria, exhibited a clear higher abundance only in fractions P-100 and P-40, with the latter fraction exhibiting the highest relative abundance (ART-C test, *p* < 0.001; Fig. 3a, Fig. S6). Bacteroidia, the most abundant class in the samples, had a significantly lower abundance in in P-100 P-60 and P-40 but was overall highly variable between cows and replicates (ART-C test, *p* < 0.05; Fig. 3a; Fig. S6).

To further assess taxa distribution, we conducted a genus-level analysis that showed that, depending on the source of the prokaryotic community (i.e., cow), bacterial genera enriched within the Gammaproteobacteria were Succinivibrionaceae, *Succinivibrio, Ruminobacter, Acinetobacter* or all together (Fig. 3b). These genera represented on average between 2.4% and 38.7% in the protozoa-containing communities P-100, P-60, and P-40 (Fig. 3b). These genera were in either lower abundance or completely absent in the protozoa-free microcosms ranging between 0% and 6.8%. The increase in Gammaproteobacteria was contrasted mainly by *Prevotella* and *Streptococcus*, which were usually the most abundant genera in the microcosms and exhibited a significant decrease across all cows in P-100, P-60, and P-40 fractions (Wilcoxon test, *p* < 0.05 Fig. 3b). The Rikenellaceae RC9 gut group, which was also highly abundant in the microcosms, also exhibited a significantly higher proportions in the presence of protozoa with this genus being consistently higher in fractions P-100 and P-60 across the cows (Wilcoxon test, *p* < 0.05 Fig. 3b).

Our results show that the presence of protozoa had a recurrent effect on the bacterial composition, regardless of its source community, particularly with regards to the enrichment of specific taxonomic lineages.

### Protozoa favor co-existence of phylogenetically related taxa

**Fig. 4:**
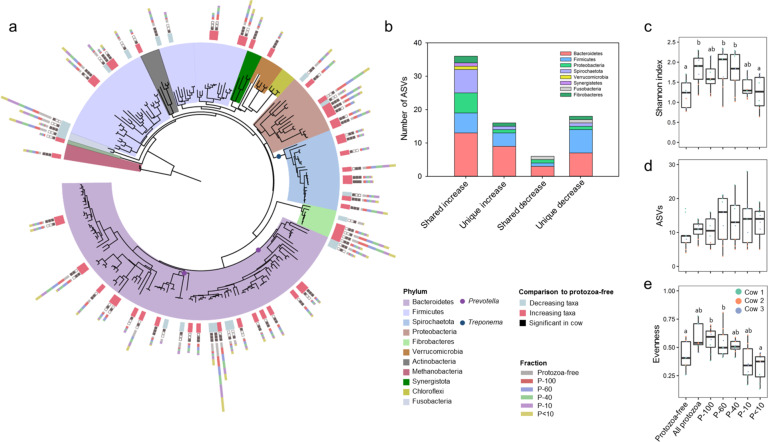
ASV distribution across the microcosms. **a** Phylogenetic tree of the ASVs that were above 0.5% relative abundance in at least one group of microcosms. Each ASV is color coded based on their phylum affiliation. The colored boxes above each ASV represent their divergence in abundance in the protozoa-containing microcosms compared to the protozoa-free microcosms (red = increasing, light blue=decreasing). The filled and empty black squares represent the cows in which the difference was observed with the filled square denoting that a difference was observed in a specific cow (*p* < 0.05). The stack bars represent the average abundance of the ASV across the different communities. The colored circles indicate the genera *Prevotella* and *Treponema* in the tree. **b** Distribution of differentially abundant ASVs in protozoa-containing microcosms compared to the protozoa-free microcosms. The stack bars denote whether the ASVs exhibited a significant increase or decrease and whether they were shared across cows or unique to one cow. **c** Shannon index **d** no. of ASVs and **e** evenness of the genus *Prevotella* across the different communities, with different letters above the boxes signifying significant differences between the groups at *p* < 0.05.

Our results of the prokaryotic taxonomic distribution so far show that protozoa had a stark effect on the microbial community composition. To assess the protozoa-mediated effect on the microbial species level, we conducted a phylogenetic analysis of ASV-level taxa (Fig. 4a). This revealed that ASV-level taxa largely reflected the taxa distribution observed at the genus level (Fig. 4a). Interestingly, we observed a significantly larger proportion of ASVs that increased in abundance in the presence of protozoa were shared between the different cows compared to ASVs that decreased (Fig. 4a, b). Only 6 out of 40 ASVs significantly decreased in protozoa-containing microcosms, which was consistent across at least two cows (3 ASVs shared by all three cows; Fig. 4b). In contrast, the number of ASVs exhibiting a higher abundance in protozoa-containing microcosms was significantly higher with 39 ASVs shared between cows and 17 shared by all cows (Fisher exact test, ASVs increasing in all cows vs. ASVs decreasing in all cows ASV; *p* < 0.001; Fig. 4a, b).

Further analysis of the distribution of taxa that are differentially abundant when incubated with protozoa, we observed that the largest proportion of ASVs belonged to *Prevotella* and *Treponema*, which were either significantly higher in abundance or exclusively found in protozoa-containing microcosms (Fig. 4a, b). Notably, *Prevotella* overall exhibited a decrease in abundance in most of the protozoa-containing microcosms (Fig. 3b). Nonetheless, we found that while overall decreasing in abundance, *Prevotella* diversity significantly increased in the presence of protozoa (protozoa communities P-100, P-60, P-40 and all-protozoa, ART-C test, *p* < 0.05; Fig. 4c–e). The increase in diversity was mostly driven by an increase in ASV richness and evenness within the genus depending on the source of the prokaryotic community (Fig. 4e), concomitant with a decrease of the dominant *Prevotella* ASVs found in the protozoa-free microcosms (Fig. 4a). A similar observation could be made within the *Treponema* genus (Fig. S7). This genus overall increased in abundance across time in all the microcosms regardless of whether these contained a protozoa community or not (Fig. S7). However, the expansion of *Treponema* in protozoa-free microcosms was limited to a small number of ASVs, while it accounted for significantly more ASVs in the all protozoa-containing microcosms (ART-C test, *p* < 0.05; Fig. 4a, b; Fig. S7). These results thus show that the presence of protozoa is directly responsible for the increase within genus diversity in these genera. Thus, in addition to the selection of specific prokaryotic lineages, the presence of protozoa may also allow for an increased co-existence of phylogenetically similar taxa within microbial communities.

## Discussion

**Fig. 5:**
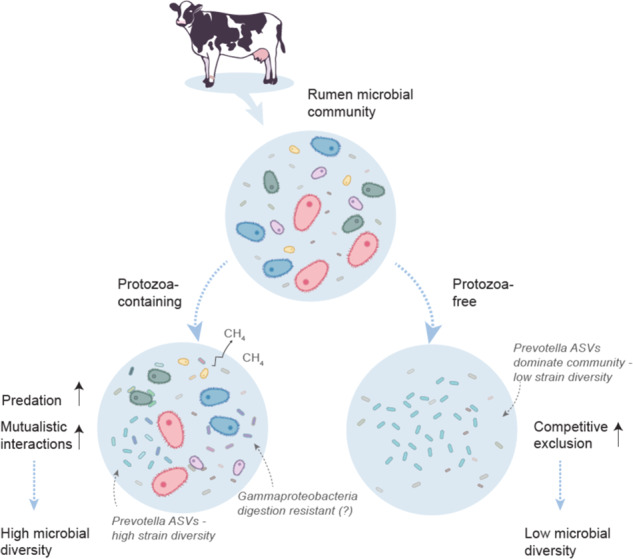
Modulatory effect of protozoa on the rumen prokaryotic community as seen in this experiment. The microbiome samples were originally taken from a mixed rumen microbial community. When the protozoa community (large cells representing all protozoa) was removed from the microbial community, prokaryotic diversity was lower than when protozoa remained in the system. This suggests that the presence of protozoa maintains diversity of the prokaryotic community of the rumen and enables the co-existence of phylogenetically related species (represented by the different shades of blue denoting *Prevotella* species). The presence of protozoa also enriches specific bacterial lineages such as Gammaproteobacteria either by metabolic mutualistic interaction or by a resistance to predation or grazing.

Host-associated microbial communities can be altered by several factors including the host species or genetics [26, 57], host lifestyle such as diet, and geography of the host [58]. In addition, within the constraint of these factors, interactions between microbial taxa can shape the community structure as well [59, 60]. While bacteria-bacteria interactions have garnered considerable attention in recent studies, the effect of the eukaryotic components of microbial communities remains largely unexplored. Here we establish an experimental system that enables us to control for the presence and absence, as well as the composition of the protozoa community. Our experimental setup allows us to show that the presence of different protozoa communities leads to significant individual changes in prokaryotic community structure as well as end-product metabolite output including methane (Fig. 5).

The protozoa-mediated enrichment of specific taxa, such as the expansion of Gammaproteobacteria families and genera, suggests metabolic interactions between bacteria and protozoa. Indeed, species of *Succinivibrionaceae* have been characterized as hydrogen utilizers, which protozoa produce in high abundance via hydrogenosome organelles or cytosolic hydrogenases [18, 61]. This observation can be extended to the concurrent increase in *Methanobrevibacter*, a methanogenic archaea genus, in which most of its known species are hydrogen utilizers (Fig. 3). Nonetheless, the rumen environment encompasses a large diversity of taxa capable hydrogen utilization, with a recent study analyzing 501 rumen genomes showing that two-third of those carry hydrogen utilization/production capabilities [62]. The expansion of Gammaproteobacteria may thus be the result of additional factors that likely confer an advantage over competing species in the presence of protozoa. Notably, Gammaproteobacteria were previously suggested to be resistant to predation in marine microbial assemblages [63]. In addition, in the rumen, Gammaproteobacteria were suggested to be underrepresented in the community that is physically associated with protozoa cells compared to the free-living prokaryotic community [64]. It was further suggested that type III, IV, and VI secretion systems, which are commonly encoded in the genome of this class, may play a role in the observed resistance to predation [63, 65]. Notably, type III secretion systems were also identified as highly abundant in Gammaproteobacteria species in the rumen environment [66]. In addition, Gammaproteobacteria genera in the rumen environment were shown to display a large variability in abundance between animals, even under similar management and diet [67, 68]. Notably, *Succinivibrionaceae* were observed to be associated with higher feed efficiency and lower methane emissions in ruminants and other foregut hosts [69, 70]. Therefore, our results may offer an explanation to such variations, where protozoa composition and abundance play a role in enriching Gammaproteobacteria, subsequent metabolic output, and animal phenotype.

In their role as microbial predators, a large body of theoretical framework as well as empirical evidence show that protozoa modulate the relationship between microorganisms by exerting top-down control on the overall structure of prey communities [9, 71–73]. Predators are often considered keystone species in an ecosystem, as they are able to impose a strong selection on prey communities even when they are found in low abundance. Bactivorous predation by protozoa is often considered of a generalist nature, where a wide breadth of feeding preference can be observed largely affecting bacterial density, but less so the overall community composition [7]. This is in contrast to selective predation, which has the potential to extinguish entire clonal populations, thus changing prokaryotic composition. In the rumen, Gutierrez observed that *Isotricha prostoma* preferentially ingested bacteria of specific morphologies [74]. In contrast, Coleman observed that *Entodinium caudatum* had no preference in bacterial prey when offered bacterial mixtures with differing proportions [75]. Based on our observation of a stark change in community composition, it is likely that selectivity in prey species exists in the rumen protozoa populations. One reason for such observation may be adaptation of prey species via increased resistance to predation or grazing such as those proposed for Gammaproteobacteria taxa [63].

Microcosm experiments as well as theoretical models previously demonstrated that exposure to predation or grazing pressure mitigates competition between species with overlapping niches in a competition-predation trade-off [71, 76, 77], ultimately leading to an increase in diversity parameters. This is in line with our findings, where the presence of protozoa in the rumen community increases diversity that is driven by both increased evenness and ASV number by the end of the experiment (Fig. 2). Our results further strengthen this notion, as we find that the presence of protozoa decreases the abundance of the most dominant ASVs in *Prevotella* or *Treponema*, concomitant with an increase ASV abundance of phylogenetically related ASVs and overall increasing within genus diversity (Fig. 4). These genera are an example of the protozoa-mediated effect in mitigating competition between phylogenetically and possibly metabolically similar taxa. Our results thus further suggest that protozoa play a central role in promoting species co-existence of species with overlapping niches in complex microbial communities. Whether species co-existence is the result of reduced competition or other causes such as increase in nutrient availability, or broad environmental modification such as oxygen scavenging, which protozoa are known to do, remains to be further evaluated. A role of promoting diversity of protozoa in host-associated communities was also proposed for the eukaryotic community in the human gut [15, 78, 79].

Interestingly, the increase in diversity parameters was generally most pronounced in fractions containing intermediate sized protozoa (P-40, Fig. 2). This observation may be interpreted as a result of a competition-predation trade-off, where protozoa-free microcosms and microcosms containing only large protozoa (P-100) represent two extreme scenarios leading to taxa extinction due to either high competition (protozoa-free) or strong predation obtained by the larger protozoa biomass in P-100. In contrast, intermediate-sized protozoa may represent an equilibrium between the competition-predation trade-offs, displaying a higher species diversity. This scenario fits prior experimental models, which show that prey diversity is maximized at intermediate predation intensity [80]. However, validating this hypothesis requires further experimentation that would include a decoupling of protozoa size from their identity [10, 76].

Our results further show that protozoa play a pivotal role in the rumen microbiome end-product output comprising VFAs and methane (Fig. 1). While the higher production of several of the quantified metabolites such as acetate and butyrate may be related to the protozoa metabolism [29], methane is likely the result of mutualistic interactions between methanogenic species and hydrogen-producing microbes. Several microbial eukaryotes form mutualistic (or commensal) relationships with prokaryotes across a wide range of environments [2, 81]. Indeed, rumen protozoa were shown to be habitat for a large methanogenic community that is physically associated with the protozoa cells [32, 34, 82]. Specifically, the *Isotricha* enriched fraction exhibited the highest methane production, even when protozoa contribution was normalized by biomass. Holotrich protozoa, such as *Isotricha*, have been previously shown to play a role in supporting methanogenesis that is likely due to the observed higher activity of their hydrogenosomes [18, 61]. The elevated methane emission was suggested to be the result of a mutualistic relationship between the hydrogen-producing protozoa and the hydrogenotrophic methanogens. Our results are in line with this observation and further show that the hydrogen-utilizing methanogenic community increased in the presence of protozoa (Fig. 3). Thus, the strong increase in methane emission measured in the presence of protozoa is likely explained by the protozoa-associated microbial community.

Many experiments studying the effect of micro-eukaryotic predators on the bacterial community use artificial prey communities that comprise only a low number of different species or a simplified bacterial community [7, 71, 83]. Here, we assessed the direct impact of the presence and absence of natural protozoa communities on their native prokaryotic communities. Accordingly, our study provides insights into natural dynamics as well as the multifaceted role of microbial eukaryotes in microbial habitats. Protozoa feed on the microbial populations, yet they also provide habitats and nutrients for mutualistic exchange to their surrounding prokaryotes. Thus, when studying the rumen microbial ecosystem, the role of cross-domain interactions between protozoa and prokaryotes need to be taken into consideration. Modulation of ciliates may bear great potential in affecting its surrounding prokaryotic community, which may also lead toward improved animal phenotypes.

## Supplementary information

Supplementary material

## Data Availability

The sequencing data were deposited into the Sequence Read Archive (SRA) of NCBI and can be accessed via the bioproject accession number PRJNA764499.
