# Supplementary material for: Protozoa populations are ecosystem engineers that shape prokaryotic community structure and function of the rumen microbial ecosystem
Source: ISME J. 2021 Dec 9;16(4):1187–97. doi: 10.1038/s41396-021-01170-y (PMC8941083; doi:10.1038/s41396-021-01170-y)
Supplement: Supplementary file 1 — Supplementary material [file 41396_2021_1170_MOESM1_ESM.pdf]

Protozoa populations are ecosystem engineers that shape prokaryotic community structure and function in the rumen ecosystem

Ronnie Solomon, Tanita Wein, Bar Levy, Shahar Eshed, Rotem Dror Veronica Reiss, Tamar Zehavi, Ori Furman, Itzhak Mizrahi, Elie Jami

Supplementary Material

Supplementary Figures

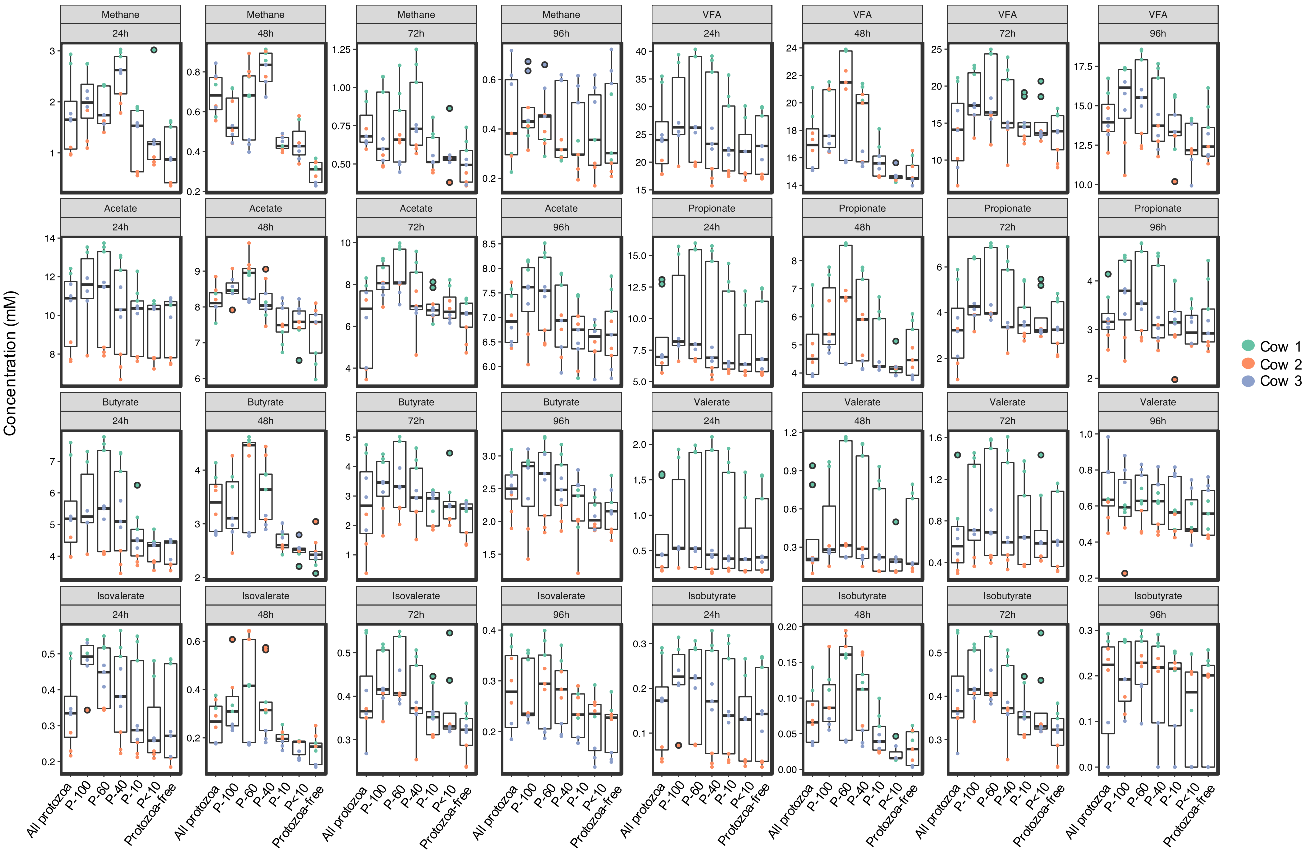

Supplementary Figure S1. Production of VFAs and methane over time. Metabolites were measured in microcosms containing the rumen microbial community incubated with different protozoa communities and protozoa-free microcosms. VFAs and methane were measured every 24h for a total of 96h. Kruskal-Wallis statistical test was performed to test for differences between the protozoa fractions and are shown in Table S1.

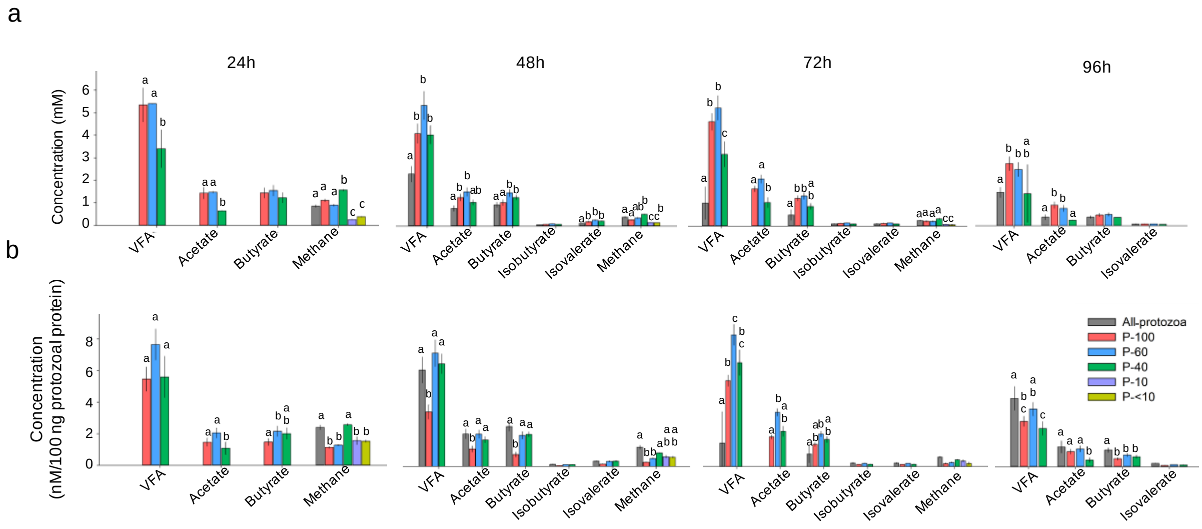

Supplementary figure S2. Average change in metabolite production in the presence of different protozoa fractions. The average was obtained by subtracting the raw quantification of metabolites in each protozoa fraction with the values obtained from the protozoa-free community of each for each cow separately with (a) representing the average increase (b) the average increase standardized by the biomass of each fraction. Only fractions and metabolites that exhibited a significant difference with the protozoa free community were assessed (Based on Table S1; *p* <0.1). Statistical analysis was performed using ART-C corrected for multiple tests with different letters above the bars denoting significance at $p < 0.05$

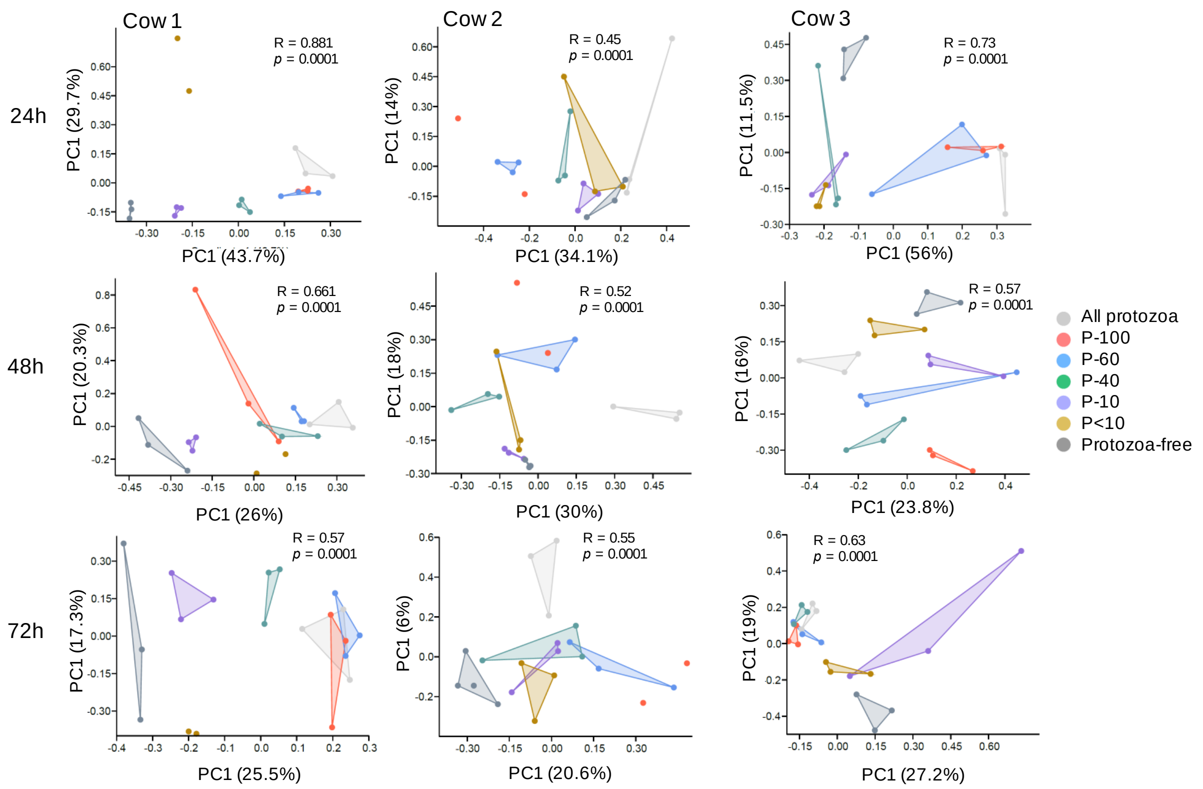

Supplementary Figure S3. Ecological structure of the prokaryotic community across time. Principal coordinate analysis (PCOA) based on pairwise Bray-Curtis distance metric of the microcosms plotted by source sample and for every day of the experiment (24h to 72h; 96h is shown in Fig. 2a). Analysis of similarity test (ANOSIM) on the overall groups was performed and displayed for each plot. This analysis shows that discrimination based on the different protozoa communities introduced to the prokaryotic communities, can be detected after 24h.

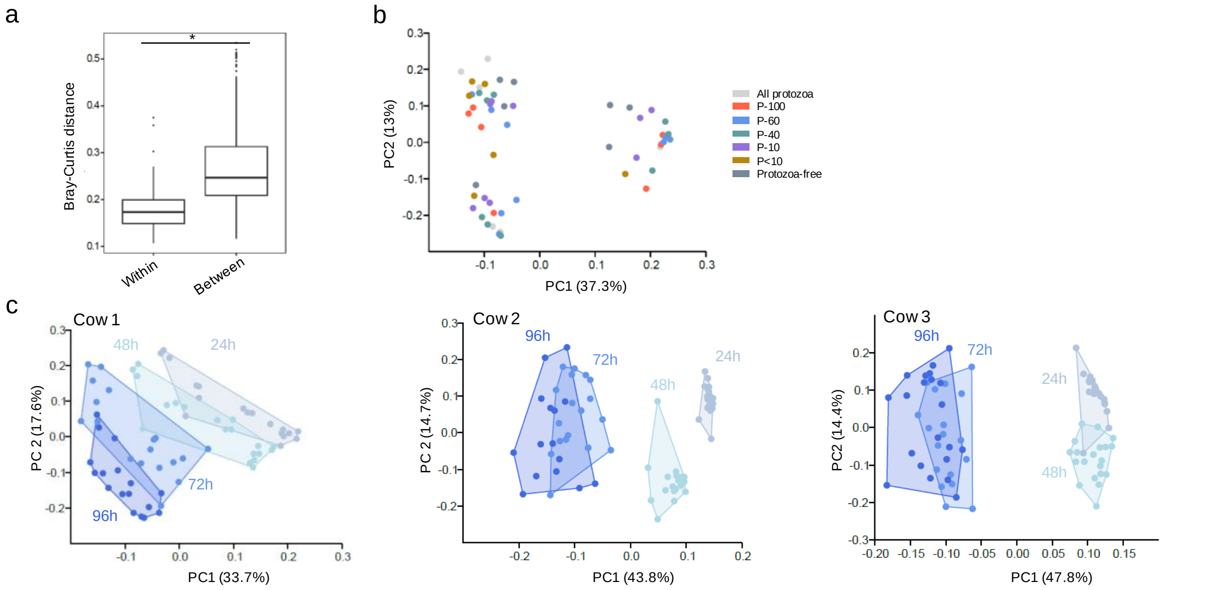

R \ p	24h	48h	72h	96h
24h		0.001	0.001	0.001
48h	0.22		0.001	0.001
72h	0.62	0.43		0.04
96h	0.88	0.71	0.34	

R \ p	24h	48h	72h	96h
24h		0.001	0.001	0.001
48h	0.86		0.001	0.001
72h	0.97	0.90		0.046
96h	0.99	0.93	0.15	

R \ p	24h	48h	72h	96h
24h		0.001	0.001	0.001
48h	0.7253		0.001	0.001
72h	0.973	0.9686		0.06
96h	0.9705	0.9586	0.1604	

Supplementary Figure S4. Effect of incubation time and cow individuality on microbiome structure. (a) Bray-Curtis distance within replicates across the microcosms compared to the distance between the replicates. The values used are the individual values obtained for each source (i.e., cow) sample. Pairwise Wilcoxon test was used to test for significance ($p < 0.001$). (b) Effect of cow individuality on prokaryotic structure. The PCOA shows 3 separate clusters representing each cow and color coded based on the protozoa community present in each sample at the end of the experiment. This demonstrates the strong effect of the origin of the rumen fluid on prokaryotic composition. (c) Principal coordinate analysis (PCOA) based on pairwise Bray-Curtis distance metric of the microcosms plotted for the different cows and colored based on the day of sampling of the microcosms after the beginning of incubation (every day from 24h - 96h). The table below each plot denotes the ANOSIM R values and their corresponding *p*-values between the different days. We can observe rapid dynamic change between the first 3 days which subsides on the 4th day, and marginally different when compared to the third day.

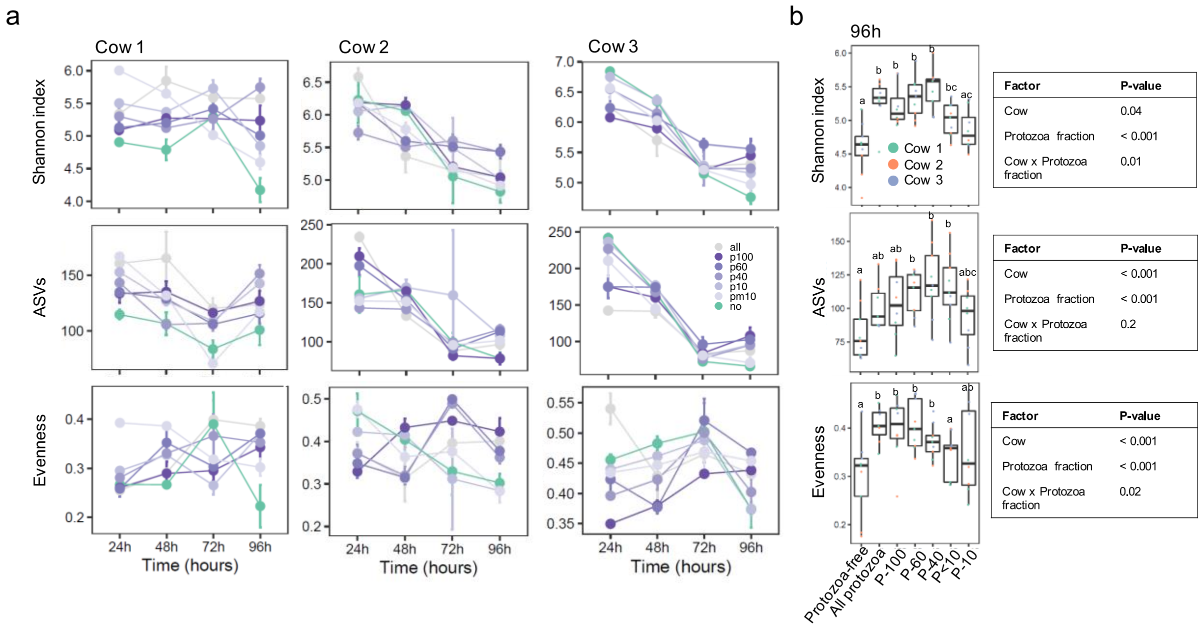

Supplementary Figure S5. Prokaryotic alpha diversity across all microcosms. (a) Alpha diversity parameters plotted for individual cows and across time from 24h to 96h including ASV richness (ASVs), Shannon index and evenness across the different microcosm groups. We observed different dynamics across the different cows which invariably lead to higher diversity parameters in microcosms containing protozoa of larger sizes (P-100, P-60, P-40), yet a trend is notable in microcosms containing small protozoa (P-10 and P<10). (b) Boxplots display the alpha diversity parameter at the end of the experiment (96h). Differences between the groups was assessed using an aligned rank transformed ANOVA (ART) procedure and *post hoc* test ART-C test, with different letters above the boxes signifying significant differences between the groups.

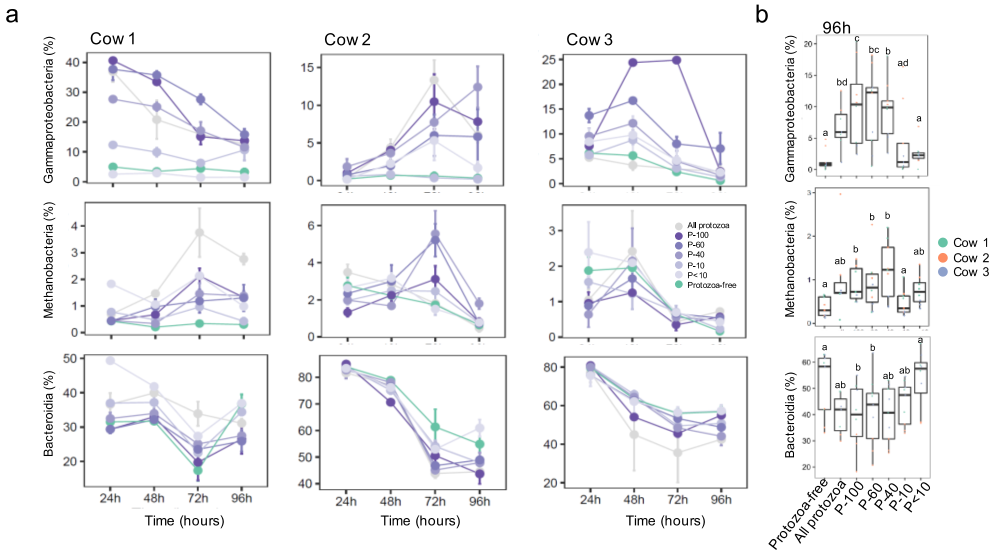

Factor	*p*-value
Cow	<0.001
Protozoa fraction	<0.001
Cow x Protozoa fraction	<0.068

Factor	*p*-value
Cow	<0.001
Protozoa fraction	<0.001
Cow x Protozoa fraction	0.07

Factor	*p*-value
Cow	<0.001
Protozoa fraction	<0.001
Cow x Protozoa fraction	0.66

Supplementary Figure S6. Relative abundance of taxonomic classes in the microcosms over time. (a) Relative abundance of class level taxa. Shown are dynamics of abundance across the different protozoa communities inoculated in the microcosms throughout the experiment (every 24h). (b) Boxplots display the relative abundance at the end of the experiment (96h). Differences between the groups was assessed using aligned rank transformed ANOVA (ART) procedure and *post hoc* test ART-C test, with different letters above the boxes signifying significant differences between the groups.

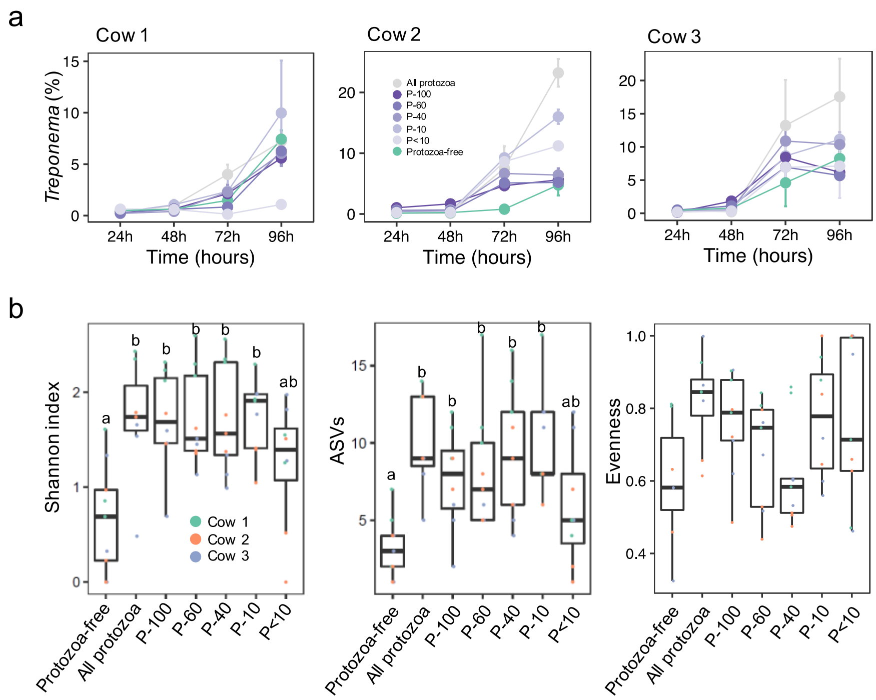

Supplementary Figure S7. Relative abundance and diversity parameters of the genus *Treponema*. (a) Relative abundance of the genus Treponema across time. This genus exhibited an increase in abundance across time, which was not significantly different between the protozoa-free microcosms and most protozoa containing microcosms except in the all-protozoa and P<10 microcosm. (b) Shannon index, ASV richness (ASVs) and evenness of the genus *Treponema* at the end of the experiment (96h) is displayed across the different microcosms. We note a significantly higher Shannon diversity and ASV richness in all the groups containing protozoa. Differences between the groups was assessed using aligned rank transformed ANOVA *post hoc* test ART-C, with different letters above the boxes signifying significant differences between the groups**.**

Supplementary Tables

Supplementary Table S1. Statistical analysis of metabolites shown in Fig.1d and S1 for the differences between the different microcosm fractions.

	***p*-value**			
**Metabolites**	**24h**	**48h**	**72h**	**96h**
Methane	<0.01	<0.01	<0.01	0.21
VFAs	0.09	<0.01	0.04	0.04
Acetate	0.08	<0.01	<0.01	0.054
Propionate	0.56	0.11	0.37	0.41
Butyrate	0.07	<0.01	0.07	0.09
Valerate	0.5	0.38	0.65	0.52
Isovalerate	0.09	<0.01	<0.01	<0.01
Isobutyrate	0.21	0.028	<0.01	0.44

Supplementary Table S2. The table shows the PERMANOVA analysis across the protozoa communities after 96h. The values on the lower left side of the table represent the F statistic and the upper right side of the table the corresponding *p*-values.

**F/*p*-value**	**All-protozoa**	**P-100**	**P-60**	**P-40**	**P-10**	**P-<10**	**Protozoa-free**
**All-protozoa**		0.25	0.13	0.31	0.03	0.03	<0.01
**P-100**	1.26		0.93	0.92	0.09	0.03	0.019
**P-60**	1.71	0.34		0.80	0.09	<0.01	0.012
**P-40**	1.15	0.30	0.45		0.09	<0.01	<0.01
**P-10**	2.35	1.78	1.98	1.87		0.14	0.25
**P-<10**	2.82	2.98	3.98	3.83	1.8		0.16
**Protozoa-free**	3.36	2.61	3.11	3.3	1.25	1.6	

Supplementary Methods

16s rRNA Data analysis

Downstream processing of the 16S rRNA data, up to the generation of the amplicon sequence variant table (ASV) was performed in QIIME v.2 [1]. Raw reads were imported to qiime platform by import command: qiime tools import --type EMPPairedEndSequences --input-path reads --output-path emp-paired-end-sequences.qza. Thereafter, reads were demultiplexed according to their conjugated barcodes by the demux command: qiime demux emp-paired --m-barcodes-file metadata.txt --m-barcodes-column BarcodeSequence --i-seqs emp-paired-end-sequences.qza --o-per-sample-sequences demux.qza --p-rev-comp-mapping-barcodes --o-error-correction-details demux_details.qza. DADA2 was applied to model and correct Illumina-sequencing amplicon errors and clustering of ASVs [2] using the command - qiime dada2 denoise-paired --i-demultiplexed-seqs demux.qza --p-trim-left-f 10 --p-trim-left-r 10 --p-trunc-len-f 150 --p-trunc-len-r 150 --o-table table.qza --o-representative-sequences rep-seqs.qza --o-denoising-stats denoising-stats.qza. Taxonomic assignment for the bacterial 16S rRNA was performed using the pre-trained classifier Silva database [3] (silva_138_16S.97) ASVs from 515F/806R region from QIIME v.2 pipeline by the command: qiime feature-classifier classify-sklearn --i-classifier Silva 138 99% OTUs from 515F/806R region of sequences.qza --i-reads rep-seqs-dn.qza --o-classification taxonomy.qza. After the generation of the ASV table, singletons/doubletons were removed and subsampling to an even depth of 4,000 reads per sample was performed for all subsequent analyses. Alpha and Beta diversity analyses were performed using was used and plotted using the PAleontological STatistics software (PAST) [4], including principal coordinate analysis (PCOA) using the Bray-Curtis dissimilarity metric and ASV richness, evenness and Shannon index. Analysis of similarity (ANOSIM) was used to test the significance of the group clustering. Distance-based redundancy analysis (DB-RDA) was performed with the capscale function in the vegan package in r [5], using the Bray-Curtis distance metric and replotted using ggplot2 [6]. Centered log ratio (CLR) for compositional data was performed using the 'composition' package in r using first the zeroreplace routine and then the clr function [7].

Aligned ranked transformed ANOVA was performed using the artool package in r using the art() and art.con() function [9].

References

1. Bolyen E, Rideout JR, Dillon MR, Bokulich NA, Abnet CC, Al-Ghalith GA, et al. Reproducible, interactive, scalable and extensible microbiome data science using QIIME 2. *Nat Biotechnol* 2019; **37**: 852–857.
2. Callahan BJ, McMurdie PJ, Rosen MJ, Han AW, Johnson AJA, Holmes SP. DADA2: High-resolution sample inference from Illumina amplicon data. *Nat Methods* 2016; **13**: 581–583.
3. Quast C, Pruesse E, Yilmaz P, Gerken J, Schweer T, Yarza P, et al. The SILVA ribosomal RNA gene database project: improved data processing and web-based tools. *Nucleic Acids Res* 2013; **41**: D590–6.
4. Hammer Ø, Harper DAT, Ryan PD. PAST: Paleontological Statistics Software Package for Education and Data Analysis. *Palaeontol Electronica* 2001; **4**: 9.
5. Oksanen J. vegan: Community Ecology Package. R package version 1.17-9.
6. Wickham, H., Chang, W., & Wickham, M. H. (2016). Package ‘ggplot2’. Create Elegant Data Visualisations Using the Grammar of Graphics. Version, 2(1), 1-189.
7. van den Boogaart KG, Tolosana-Delgado R. ‘compositions’: A unified R package to analyze compositional data. Comput Geosci 2008; 34: 320–338.
8. Wobbrock JO, Findlater L, Gergle D, Higgins JJ. The aligned rank transform for nonparametric factorial analyses using only anova procedures. Proceedings of the SIGCHI Conference on Human Factors in Computing Systems. 2011. Association for Computing Machinery, New York, NY, USA, pp 143–146.
9. Elkin LA, Kay M, Higgins JJ, Wobbrock JO. An Aligned Rank Transform Procedure for Multifactor Contrast Tests. 2021; arXiv 2102: 11824 .
